# Immunohistochemical study of tumor necrosis factor-alpha(TNF- α) expression in lung, liver, and spleen during aspergillosis infection

**DOI:** 10.1186/1471-2164-15-S2-P71

**Published:** 2014-04-02

**Authors:** Batol Imran Dheeb

**Affiliations:** 1 Biology Department, College of Science, Iraqia University, Baghdad, Iraq

## Background

Tumor Necrosis Factor-alpha(TNF-α) is important proinflammatory cytokines that is produced simultaneously and share a common spectrum of biologic activities. Aspergillosis is a common and devastating complication of immunosuppression. TNF-alpha is an important proximal signal in murine invasive aspergillosis and plays a major role in host defense and regulation of the immune response. The use of TNF antagonists is associated with an increased incidence of opportunistic infections such as aspergillosis [1].

## Materials and methods

TNF-α were investigated in BALB/c mice infected intravenously with 5×106 virulent Aspergillus fumigatus conidia by using immunohistochemistry technique (IHC) ImmunoCruzTM mouse ABC staining system (sc-2017). Seven groups of animals were studied including control group (mice not infected with A. fumigatus). These groups were used to study the expression in three separated periods 7, 14, and 21 day post infection.

## Results

Expression of TNF-α in the three organs were shown in the nuclei of the tissue cells and detected by IHC technique. Depending on the scoring system used for the TNF-α, two parameters were dependent; the intensity of the staining of the nuclei and the percentage of the cells giving positive expression. The intensity of the nuclei of the stained cells was negative if there is no expression. Expression was found positive in all the studied organs, but different in score and intensity, in lung over expression (score 3+) in 7, 14 and 21 days post infection with moderate staining at 7 day and intense staining at 14, 21 day respectively, in liver expression was (score 2+, score 2+, and 3+) with moderate staining at 7 and 14 day post infection while there is intense staining at 21 post infection. The expression in the spleen was (score1+) with light staining at the period between 7 to 14 day then increase at 21 day reach to score 2+ with moderate staining. TNF-α expression in all studied sample compared with the positive control. Results show significant difference (P value >0.01) in the expression between the studied organs. Correlation between the TNF-α expression and number and size of lesion in the infected tissues were studied statistically and the study revealed that there was a significant relationship between the immunoreactive cell and its intensity with number and size of lesion in the same tissue (P value >0.01).

## Conclusions

TNF-α expression levels was time-dependent increased and related with the progress of infection that can significantly enhance resistance to A. fumigatus in BALB/c mice.
